# In vivo detection and treatment of ischemia-induced cardiac apoptosis using an MRI-detectable molecular probe and an alpha-adrenergic receptor agonist

**DOI:** 10.1186/1532-429X-14-S1-P67

**Published:** 2012-02-01

**Authors:** Justin Lam, Ildiko Toma, Yongquan Gong, Paul C Simpson, Robert C Robbins, Phillip C Yang, Rajesh Dash

**Affiliations:** 1 Cardiovascular Medicine, Stanford University, Stanford, CA, USA; 2 Cardiac Surgery, Stanford University Medical Center, Stanford, CA, USA; 3 Medicine and Cardiology, University of California, San Francisco, CA, USA; 4 Medicine and Cardiology, San Francisco VA Medical Center, San Francisco, CA, USA

## Summary

A novel anti-apoptotic alpha-adrenergic agonist preserves left ventricular ejection fraction in mice following myocardial infarction. This therapeutic effect is able to be detected and quantified non-invasively, using T2* signal loss assessment of an Annexin-SPIO molecular apoptosis probe.

## Background

Myocardial infarction (MI) leads to cardiomyopathy through a combination of programmed cell death (re: apoptosis) and necrotic cell death. The relative contribution of apoptosis to ischemic cardiomyopathy and the effect of targeting apoptosis specifically to prevent MI-induced damage is unknown. Our laboratory previously developed and validated an in vivo, MRI-detectable apoptosis probe. Annexin-V (ANX), which binds to cells in the earliest stages of apoptosis, was conjugated to superparamagnetic iron oxide (SPIO) nanoparticles, allowing for the non-invasive detection of early apoptotic cell populations (ANX-SPIO r1: 8.6 ± 0.61 mM-1 s-1 and r2: 326 ± 16 mM-1 s-1). To test the effect of apoptosis reversal in an MI model, we employed A61603 (A6), an α1-adrenergic receptor agonist, which was previously shown to rescue cardiac cells from Doxorubicin-induced cardiac apoptosis through activation of the cardio-protective ERK pathway. Our hypothesis is that A6 therapy will protect against MI-induced cardiomyopathy, and that cardiac MRI of systemic ANX-SPIO will detect and longitudinally monitor this therapeutic effect in vivo.

## Methods

Mice underwent MI (via LAD ligation) along with a subcutaneous pump implant that delivered A6 or vehicle (VEH) solution at a rate of 10 ng/kg/day over a course of two weeks. Cardiac MRI (CMR) was performed at 2 days, 1 week, and 2 weeks post MI. ANX-SPIO was delivered via tail vein one day prior to CMR to simultaneously assess left ventricular function and apoptosis (using T2* signal loss as a marker of apoptotic activity) in vivo.

## Results

**Figure 1:**
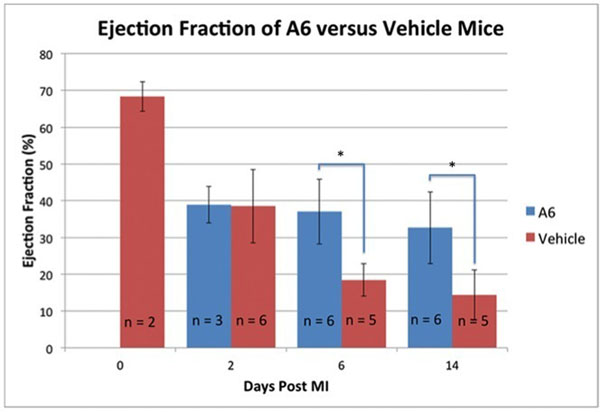
Graph of EFs for A6-treated vs VEH-treated mice over a two week period. Note the significant preservation of EFs in A6-treated hearts at 1 and 2 weeks.

**Figure 2:**
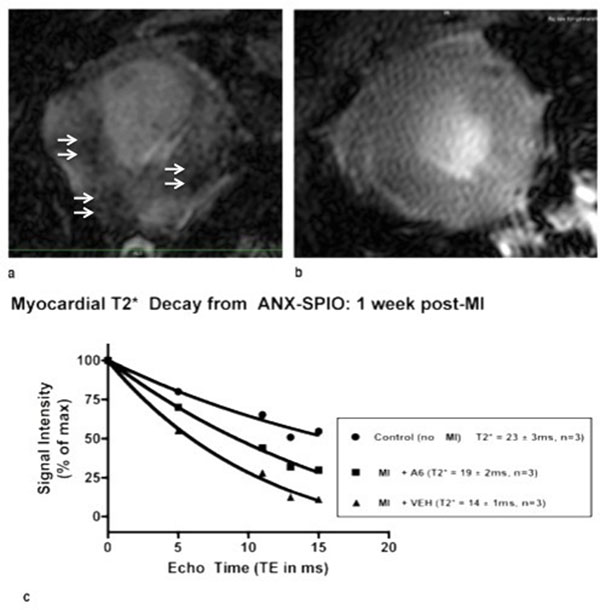
a) CMR images of an infarcted mouse heart showing T2* signal loss from ANX-SPIO; b) CMR of A6-treated, infarcted mouse heart showing a visibly reduced degree of T2* signal loss, compared to VEH control hearts; c) T2* signal loss curves for control animals, as well as MI+A6-treated and MI+VEH hearts. The steeper decay curve of MI+VEH hearts reflects the increased apoptosis and subsequent ANX-SPIO uptake, compared to A6-treated hearts.

Although A6-treated (39±5%, n=3) and VEH-treated (38±10%, n=6) mice exhibited identical ejection fractions (EFs) 2 days post-MI, A6-treated mice exhibited significantly (p<0.05) higher ejection fractions (EFs) versus their VEH-treated counterparts at both 1 week (A6, n=6: 37±9%; VEH, n=5: 18±4%) and 2 weeks (A6, n=5: 33±10%; VEH, n=6: 14±7%) post-MI (Figure 1). Upon T2* quantification, A6-treated mice showed significantly (p<0.05) less T2* signal loss after ANX-SPIO delivery compared to VEH-treated mice at 1 week post MI (A6 T2*: 19±2ms; VEH T2*: 14±1, n=3), reflecting less myocardial uptake of ANX-SPIO and therefore less cardiac cell apoptosis in A6-treated hearts (Figure 2).

## Conclusions

These results suggest that cardiomyocyte apoptosis is a prominent contributor to the functional impairment of ischemic cardiomyopathy and that A6-mediated cardioprotection from MI-induced apoptosis preserves cardiac function. Moreover, ANX-SPIO can non-invasively detect and monitor A6’s therapeutic effect longitudinally.

## Funding

NIH-NHLBI K08 (RD), R01 (PY).

